# Storage Stability of Premixed Calcium Silicate-Based Sealers Under Different Temperature and Humidity Conditions

**DOI:** 10.3390/ma19173787

**Published:** 2026-09-06

**Authors:** Sang Won Kwak, Hyeonu Jo, Asgeir Sigurdsson, Hyeon-Cheol Kim

**Affiliations:** 1 Department of Conservative Dentistry, School of Dentistry, Dental Research Institute, Dental and Life Science Institute, Pusan National University, Yangsan 50612, Republic of Korea; endokwak@pusan.ac.kr (S.W.K.); hy5959546@gmail.com (H.J.); 2 Department of Endodontics, New York University College of Dentistry, New York, NY 10010, USA; asgeir.sigurdsson@nyu.edu

**Keywords:** premixed calcium silicate sealer, bioceramic sealer, storage stability, temperature, relative humidity, flow

## Abstract

Premixed calcium silicate-based sealers (CSSs) require moisture for hydration and setting, raising concerns regarding the influence of environmental storage conditions on their physicochemical properties. This study evaluated the effects of storage temperature and relative humidity on the flow and setting time of premixed CSSs. Four premixed CSSs (One-Fil, CeraSeal, WellRoot ST, and EndoSeal MTA) were stored in their original syringes under nine environmental conditions generated by combining three temperatures (4 °C, 25 °C, and 50 °C) and three relative humidity levels (30%, 50%, and 80%) for 48 h. Flow and setting time were subsequently measured using standardized test procedures. Flow data were analyzed using a permutation-based two-way ANOVA, whereas setting time data were analyzed using nonparametric methods (α = 0.05). All tested CSSs maintained flow values above the minimum ISO requirement (17 mm) under all storage conditions. Significant differences in flow were identified among the tested storage conditions (*p* < 0.001), and the magnitude of these changes varied among the materials. WellRoot ST and CeraSeal generally exhibited greater flow than the other tested sealers. One-Fil exhibited the shortest setting times, whereas EndoSeal MTA exhibited the longest. Significant differences in setting time were also observed among storage conditions, although the magnitude and pattern of these differences were material-dependent. Overall, environmental storage conditions significantly influenced the physicochemical behavior of premixed CSSs, particularly flow. These findings improve the current understanding of the short-term storage stability of premixed CSSs and suggest that environmental conditions should be considered when storing and handling these materials.

## 1. Introduction

Calcium silicate-based sealers (CSSs), often referred to as bioceramic sealers, have been increasingly adopted in contemporary endodontic practice due to their favorable physicochemical and biological properties [1,2,3]. These materials exhibit excellent biocompatibility and low cytotoxicity, promoting a favorable tissue response when in contact with periapical tissues [4,5]. In addition, their bioactivity allows for the formation of hydroxyapatite during the setting process, contributing to chemical bonding with dentin and potentially enhancing the long-term sealing ability of the root canal system [2,6,7]. Other reported advantages include dimensional stability, slight expansion upon setting, and inherent antimicrobial properties associated with their high pH during hydration [8,9]. Furthermore, the availability of premixed, ready-to-use formulations in syringe delivery systems has improved clinical convenience and minimized technique sensitivity compared with conventional sealers [1,9,10]. Unlike powder–liquid hydraulic cements that are mixed immediately before use, premixed sealers are stored for extended periods within their delivery syringes before clinical application. Consequently, maintaining storage stability throughout distribution and clinical storage is an important prerequisite for predictable performance.

Despite these advantages, CSSs possess distinct setting characteristics that differentiate them from traditional resin-based or zinc oxide–eugenol-based materials [8,11]. Their setting reaction is hydration-dependent, requiring the presence of moisture to initiate and complete the process [11]. While this property is beneficial within the moist environment of the root canal, it also raises concerns regarding the potential influence of environmental factors—particularly temperature and humidity—during storage and handling before clinical use.

In clinical settings, storage conditions for dental materials can vary widely depending on geographic location, seasonal climate changes, and differences in storage environments within dental clinics and hospitals. Exposure to different temperature and relative humidity conditions during transportation or storage may alter the physicochemical behavior of premixed CSSs before clinical use. Such environmental exposure could potentially influence material flow and setting behavior, thereby affecting their handling characteristics during root canal obturation.

Previous studies investigating premixed calcium silicate-based sealers have primarily focused on their physicochemical properties, biocompatibility, sealing ability, and clinical performance under standardized laboratory conditions [2,3,12,13]. Likewise, the influence of environmental factors has been investigated mainly in relation to the setting behavior or thermal exposure of calcium silicate materials rather than the combined effects of storage temperature and relative humidity on premixed sealers. Consequently, little information is available regarding how combined temperature and relative humidity conditions during storage influence the physicochemical properties of commercially available premixed CSSs before clinical use [13].

Although manufacturers provide recommended storage conditions, independent experimental evidence regarding the behavior of premixed CSSs under different environmental storage conditions remains limited. In particular, the combined influence of storage temperature and relative humidity has not been systematically characterized. Given the global distribution and increasing clinical reliance on these materials, a clearer understanding of their short-term behavior following exposure to different storage environments may contribute to more predictable material handling and performance.

Therefore, this study aimed to investigate the effects of storage temperature (4 °C, 25 °C, and 50 °C) and relative humidity (30%, 50%, and 80%) on the flow and setting time of selected CSSs. By simulating a broad range of environmental conditions that may be encountered during transportation and clinical storage, this study evaluated the short-term storage stability of premixed CSSs before clinical use. The null hypothesis was that the tested storage conditions would have no significant effect on the flow and setting time of the CSSs.

## 2. Materials and Methods

### 2.1. Sample Size Calculation

Sample size was determined based on preliminary pilot data assessing differences in flow and setting time among storage conditions. A power analysis was performed using G*Power 3.1 (Heinrich Heine University, Düsseldorf, Germany), assuming an effect size of 0.40, a significance level (α) of 0.05, and a statistical power (1 − β) of 0.80. The minimum required sample size was calculated to be *n* = 5 specimens per group, and five specimens were therefore included in each experimental condition.

### 2.2. Materials and Group Designation

Four commercially available premixed CSSs were included in this study: One-Fil (Mediclus, Cheongju, Republic of Korea), CeraSeal (Meta Biomed, Cheongju, Republic of Korea), WellRoot ST (Vericom, Chuncheon, Republic of Korea), and EndoSeal MTA (Maruchi, Wonju, Republic of Korea). All tested materials are intended for conventional orthograde root canal obturation and are therefore classified as Type 1 endodontic sealing materials according to ISO 6876:2025 [14]. The composition, manufacturer, and batch number of each material are presented in Table 1. All materials were used within their expiration dates and handled according to the manufacturers’ instructions. The sealers were maintained in their original commercially packaged syringes throughout the storage period, with the syringe caps kept in place.

### 2.3. Storage Conditions

To evaluate the influence of environmental conditions, all materials were stored in a programmable temperature–humidity chamber (TH-G-408; Jeio Tech, Seoul, Republic of Korea), which allows precise control of temperature (± 0.5 °C) and relative humidity (± 3%). The experimental conditions consisted of three temperature levels (4 °C, 25 °C, and 50 °C) combined with three relative humidity (RH) levels (30%, 50%, and 80%), yielding nine distinct storage environments. Each material, in its original syringe form, was stored under the assigned conditions for 48 h before testing. A storage period of 48 h was selected to simulate short-term environmental exposure that may occur during transportation, distribution, or temporary storage before clinical use. The selected temperature and relative humidity combinations were intended to represent a broad range of environmental conditions. The materials were removed from the environmental chamber immediately before testing and allowed to equilibrate to the testing conditions only for the minimum time required for specimen preparation.

### 2.4. Experimental Design

**Figure 1:**
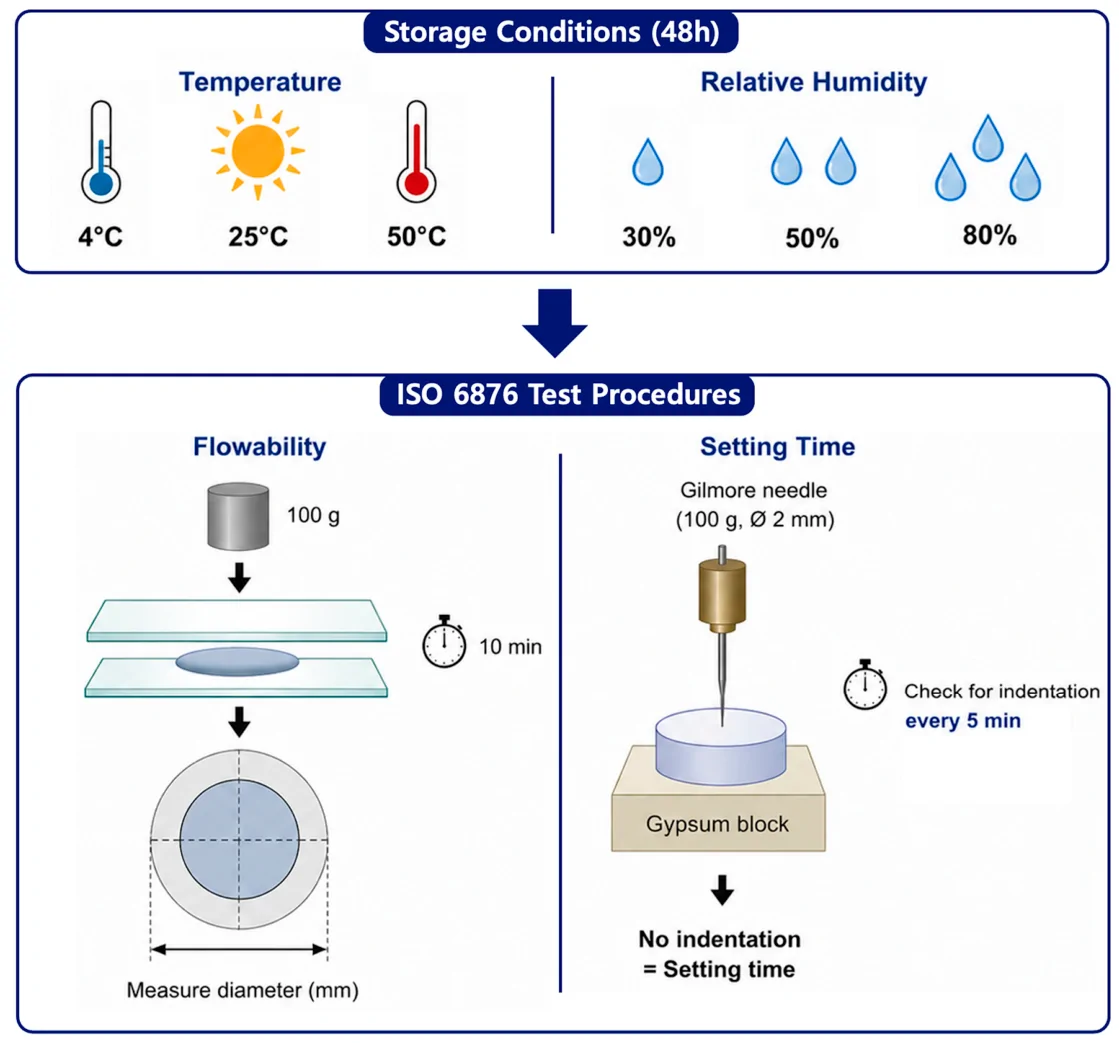
Experimental design and testing procedures. Four premixed calcium silicate-based sealers were stored in their original syringes for 48 h under nine environmental conditions generated by combining three storage temperatures (4 °C, 25 °C, and 50 °C) with three relative humidity (RH) levels (30%, 50%, and 80%). After storage, flow was evaluated by measuring the diameter of the compressed sealer specimen after applying a 100-g load for 10 min, whereas setting time was determined using a Gilmore needle (100 g, 2 mm tip diameter), with indentation assessed initially at 20-min intervals and subsequently at 5-min intervals as the material approached its expected setting time.

Immediately after completion of the storage period, all specimens were tested to minimize additional environmental exposure before measurement (Figure 1). All tests were conducted under controlled laboratory conditions, and ambient temperature and relative humidity were recorded at the time of testing. The experimental procedures were originally designed and performed according to ISO 6876:2012 [15], which was the applicable protocol adopted for this study. ISO 6876:2025 [14], published subsequently, introduced a revised classification and modifications to the setting-time test procedure. Accordingly, the procedures used in the present study are described explicitly below rather than being presented as compliant with the current ISO 6876:2025 setting-time protocol.

### 2.5. Flow Test

Flow was measured according to ISO 6876:2012 [15], which specifies a minimum flow diameter of 17 mm. A glass plate with a thickness of 5 mm was placed on an analytical balance (A&D FX-200i; A&D Co., Tokyo, Japan; readability 0.001 g), and the balance was zeroed. A volume of 0.05 mL sealer was carefully dispensed onto the center of the plate to minimize initial contact area. A second glass plate of identical dimensions was then placed over the material, and a 100 g load was centrally applied.

At the moment the load was applied, timing was initiated using a stopwatch and maintained for 10 min. After removal of the load, the compressed sealer formed a circular disc, the diameter of which was measured using a digital caliper (Mitutoyo Corp., Kanagawa, Japan; accuracy ± 0.02 mm, resolution 0.01 mm). The maximum and minimum diameters were recorded, and when the difference between these values was ≤1 mm, their mean value was calculated and recorded as the flow value.

### 2.6. Setting Time

Setting time was measured based on ISO 6876:2012, which was the standard adopted when the experimental study was designed and conducted [15,16]. Gypsum blocks were conditioned by immersion in water maintained at 37 °C for at least 24 h. A circular metal mold with an internal diameter of 10 mm and a thickness of 2 mm was positioned and secured onto the surface of the gypsum block, and the sealer was placed into the mold.

Sealer samples were placed in an incubator (TH-G-408, Jeio Tech, Seoul, Republic of Korea) at 37 °C and 95% relative humidity. A Gilmore apparatus equipped with a flat-ended indenter (100 ± 0.5 g, tip diameter 2.0 ± 0.1 mm) was used. The indenter was carefully placed vertically on the surface of the CSS specimen. Indentation assessments were initially performed at 20-min intervals and subsequently every 5-min interval as the material approached its expected setting time. Setting time was recorded when the indenter no longer produced a visible indentation on the specimen surface.

### 2.7. Statistical Analysis

All data were analyzed using IBM SPSS Statistics Ver. 29 (IBM, Armonk, NY, USA). Normality was assessed using the Shapiro–Wilk test, and homogeneity of variance was evaluated using Levene’s test. Flow data showed a significant deviation from normality (Shapiro–Wilk test, *p* < 0.001), whereas homogeneity of variance was satisfied (Levene’s test, *p* = 0.334). Therefore, a permutation-based two-way analysis of variance (ANOVA) was used for flow data, with material type (four sealers) and storage condition (nine temperature–relative humidity combinations) defined as the two independent factors. The effects of material type, storage condition, and their interaction were evaluated. Pairwise comparisons among storage conditions within each material were subsequently performed with adjustment for multiple comparisons.

Setting time data did not satisfy the assumption of normality and were analyzed separately for each material using the Kruskal–Wallis test across the nine storage conditions, followed by Dunn’s multiple-comparison test with Bonferroni adjustment. No independent effects or interactions between temperature and relative humidity were inferred from the setting-time analysis. Statistical significance was set at α < 0.05.

## 3. Results

### 3.1. Flow

**Figure 2:**
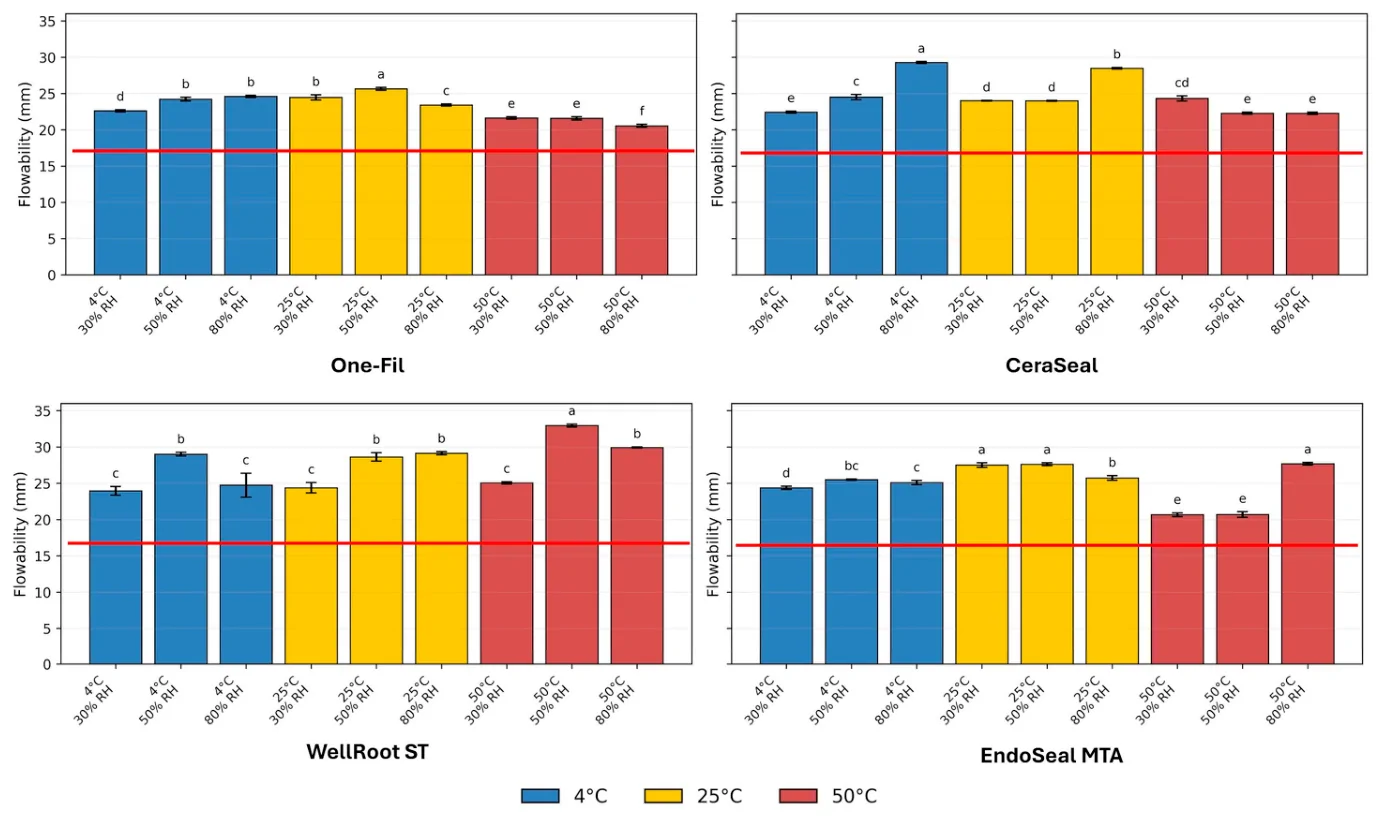
Flow of the tested calcium silicate-based sealers after storage under different temperature and relative humidity (RH) conditions. Bars represent mean values, and error bars indicate standard deviations. Storage conditions are expressed as temperature (°C) and relative humidity (%). The horizontal red line represents the minimum flow requirement (≥17 mm) specified for Type 1 endodontic sealing materials by ISO 6876. Different lowercase letters indicate statistically significant differences among storage conditions within each material (*p* < 0.05; permutation-based two-way ANOVA followed by adjusted pairwise comparisons).

The mean flow values under the different storage conditions are presented in Figure 2. All tested CSSs exhibited flow values above the minimum requirement of 17 mm specified by ISO 6876:2012 [15] and ISO 6876:2025 [14] for Type 1 endodontic sealing materials.

Permutation-based two-way ANOVA, with material type and storage condition (nine temperature–relative humidity combinations) as independent factors, demonstrated significant effects of material type and storage condition on flow (*p* < 0.001), together with a significant material × storage-condition interaction (*p* < 0.001). This interaction indicates that the pattern of change in flow across the nine storage conditions differed among the tested materials.

Overall, CeraSeal and WellRoot ST showed relatively greater flow values than One-Fil and EndoSeal MTA; however, the magnitude and direction of changes across storage conditions were material-dependent. The highest flow value was observed for WellRoot ST stored at 50 °C/50% relative humidity. Post-hoc comparisons identified significant differences among several storage conditions within each material, as indicated by different lowercase letters in Figure 2.

### 3.2. Setting Time

**Figure 3:**
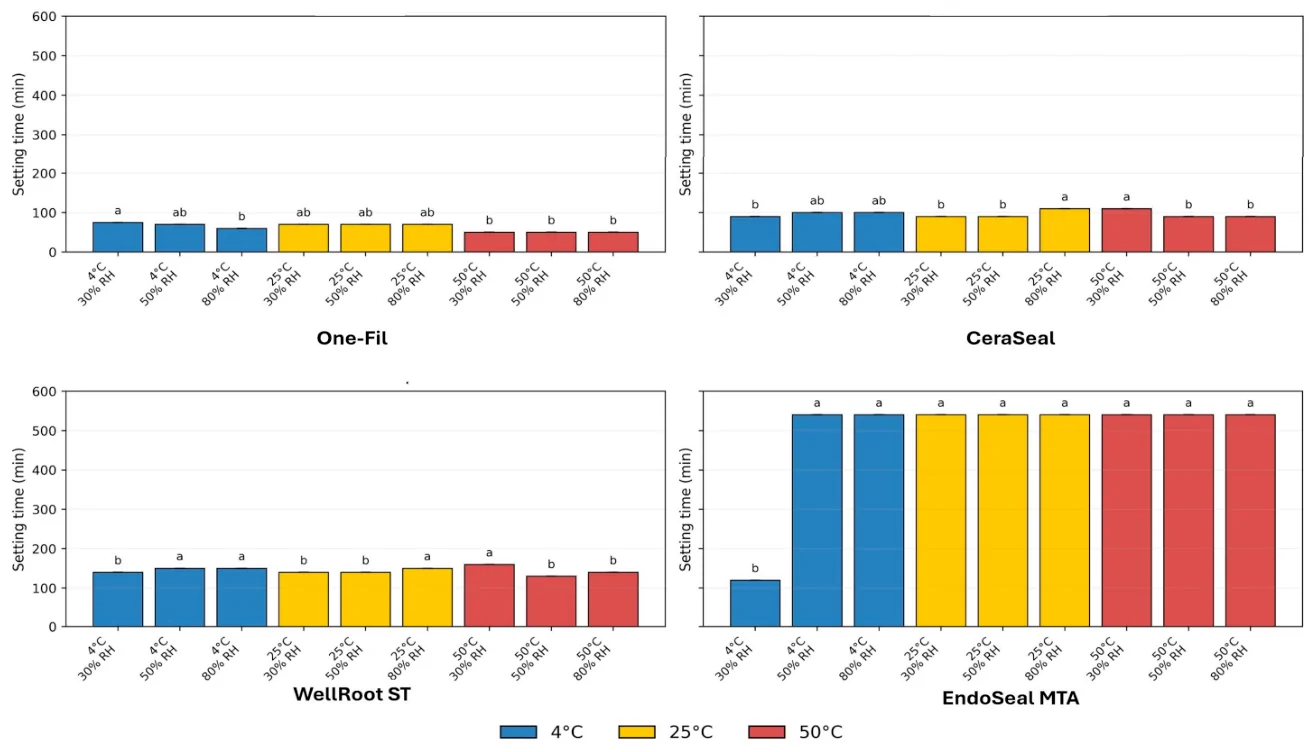
Setting time of the tested calcium silicate-based sealers after storage under different temperature and relative humidity (RH) conditions. Bars represent mean values, and error bars indicate standard deviations. Storage conditions are expressed as temperature (°C) and relative humidity (%). Different lowercase letters indicate statistically significant differences among storage conditions within each material (*p* < 0.05; Kruskal–Wallis test followed by Dunn’s multiple-comparison test with Bonferroni adjustment). Within each experimental group, all five specimens exhibited identical setting-time measurements at the predefined measurement resolution, resulting in a standard deviation of zero; therefore, error bars are not visible.

Setting times of the tested sealers under the nine storage conditions are presented in Figure 3. Kruskal–Wallis analysis demonstrated significant differences in setting time among the nine storage conditions for each tested material (*p* < 0.001). Subsequent Dunn’s multiple-comparison tests with Bonferroni adjustment identified significant differences among specific storage conditions, as indicated by different lowercase letters in Figure 3.

The pattern of setting time changes across storage conditions differed among the tested sealers. Overall, One-Fil showed shorter setting times, whereas EndoSeal MTA showed longer setting times under most storage conditions. Notably, EndoSeal MTA exhibited a setting time of 120 min at 4 °C/30% relative humidity, whereas substantially longer setting times were recorded under most of the other tested conditions.

Within each material × storage-condition group, all five specimens yielded identical setting-time measurements at the predefined measurement resolution, resulting in a standard deviation of zero. Consequently, error bars are not visible for any of the groups in Figure 3.

## 4. Discussion

The present study investigated the short-term storage stability of premixed CSSs under different temperature and relative humidity conditions. The null hypothesis was rejected because significant differences in flow and setting time were observed among the tested storage conditions. For flow, the significant material × storage-condition interaction further indicated that the response to environmental storage was material-dependent. Changes in flow were observed across multiple storage conditions, whereas setting-time responses showed different material-dependent patterns. These findings indicate that the physicochemical response of premixed CSSs to short-term environmental exposure cannot necessarily be generalized across different commercial formulations.

Premixed CSSs contain calcium silicate particles suspended within a nonaqueous carrier vehicle [17]. Unlike conventional powder–liquid calcium silicate cements, these materials are supplied ready for use and initiate setting upon exposure to moisture. This characteristic simplifies clinical procedures but simultaneously raises concerns regarding storage stability because inadvertent moisture exposure may trigger premature hydration reactions [2,11,18]. Temperature may additionally influence reaction kinetics and the rheological behavior of the carrier phase. Therefore, differences in environmental storage conditions could alter the physical state of premixed CSSs before clinical application, although the mechanisms responsible for the observed changes were not directly investigated in the present study.

Among the evaluated properties, flow showed marked differences among storage conditions. Flow is an important physicochemical property of root canal sealers because it contributes to their distribution within the root canal system [8,19,20,21]. However, penetration into anatomical irregularities, accessory canals, and dentinal tubules should not be attributed to viscosity or flow alone. Interfacial behavior is also influenced by material surface tension, substrate surface energy, wettability, contact angle, and sealer–dentin interactions [22,23,24]. Therefore, the differences in flow observed in the present study should primarily be interpreted as changes in the bulk rheological behavior and handling characteristics of the materials rather than as direct evidence of differences in their ability to penetrate complex root canal anatomy.

The material-dependent differences in flow may be associated with compositional and microstructural differences among the tested sealers. Premixed CSSs differ in the proportions and characteristics of calcium silicate phases, particle size and distribution, radiopacifying agents, nonaqueous carrier vehicles, and proprietary additives [16,17,25]. These components may influence particle–particle interactions, liquid-phase mobility, hydration kinetics, and consequently the rheological behavior of the material. Temperature changes may alter carrier viscosity and molecular mobility, whereas exposure to environmental moisture may potentially affect moisture-sensitive components or initiate limited hydration if moisture reaches the material through the delivery system. Because neither rheological parameters nor moisture uptake was directly measured in the present study, these proposed mechanisms remain speculative and require further investigation.

An important finding was that the pattern of flow changes across the nine storage conditions differed among the four materials, as demonstrated by the significant material × storage-condition interaction. This finding should not be interpreted as an independent temperature × relative humidity interaction because temperature and relative humidity were combined into a single storage-condition factor in the statistical analysis. Rather, it indicates that individual sealer formulations responded differently to the tested environmental combinations. This material-specific response further supports the importance of considering formulation when evaluating the environmental stability of premixed CSSs.

A noteworthy finding of the present study is that all tested sealers maintained flow values substantially above the minimum requirement specified by ISO 6876:2025 under all storage conditions. Although statistically significant differences were observed among storage conditions, these changes did not reduce flow below the minimum threshold. Thus, the statistical differences observed in flow should not be interpreted as failure to satisfy the ISO flow requirement. Their potential relevance may instead relate primarily to differences in handling behavior. The present findings therefore suggest a degree of tolerance of these materials to the short-term environmental exposures evaluated in this study, although they should not be extrapolated to prolonged or repeated exposure.

Setting time also differed significantly among the nine storage conditions for each material; however, the patterns were not analyzed as independent effects of temperature and relative humidity. The setting-time response varied among the tested materials and storage conditions. These differences may reflect differences in formulation, hydration kinetics, carrier systems, particle characteristics, and additives; however, the present experimental design does not permit identification of the specific compositional factor responsible for these differences.

A particularly notable finding was the setting time of 120 min recorded for EndoSeal MTA after storage at 4 °C/30% relative humidity, which was substantially shorter than that observed under most of the other tested conditions. All five specimens in this group yielded the same setting-time value at the predefined measurement resolution, indicating that the finding was reproducible within the experimental conditions used. Nevertheless, the mechanism underlying this unexpected response cannot be determined from the present data. EndoSeal MTA differs compositionally from the other tested sealers, and formulation-dependent hydration behavior may have contributed to this response. Further studies incorporating chemical and microstructural characterization are required before a mechanistic explanation can be established.

Although all tested sealers belong to the same general category of premixed calcium silicate materials, material-specific responses were observed. As discussed above, compositional and microstructural differences among formulations may contribute to these responses [17,25,26]. Accordingly, the present results should not be used to rank the tested products or to identify a universally superior material. Rather, they demonstrate that storage-associated changes are formulation-dependent [25,26,27]. Therefore, storage recommendations should primarily follow the instructions provided for each product rather than be generalized across all CSSs.

The clinical implications of the present findings are particularly relevant given the global distribution of endodontic materials. Climatic conditions vary substantially among countries and regions. Materials may encounter markedly different temperature and relative humidity conditions during transportation, distribution, temporary storage, and routine clinical use. The present study does not establish a specific “optimal” temperature–humidity combination; rather, it demonstrates that short-term exposure to different environmental conditions can produce measurable, material-dependent changes in flow and setting behavior. Clinicians should therefore follow manufacturer-recommended storage conditions and avoid unnecessary exposure of premixed CSSs to extreme environmental conditions. This consideration may be particularly relevant in regions with high ambient temperature and relative humidity or substantial seasonal variations in environmental conditions.

The present study has several limitations. First, the storage period was limited to 48 h and may not fully represent long-term storage conditions encountered in clinical practice. Second, only flow and setting time were evaluated. Other clinically relevant properties, including film thickness, solubility, dimensional stability, ion release, pH, and bond strength, may also be affected by environmental exposure [13]. Third, laboratory storage conditions may not completely reproduce the repeated temperature and humidity fluctuations encountered during material transportation, distribution, and clinical storage. In addition, this study did not quantify moisture uptake through the syringe system during storage. Furthermore, rheological properties, surface tension, wettability, and chemical or microstructural changes were not directly evaluated; therefore, definitive structure–property relationships cannot be established from the present findings.

An additional limitation concerns the setting-time methodology. Setting time was evaluated using a procedure based on ISO 6876:2012 [15], which was the standard adopted when the study was designed and conducted. Indentations were initially performed at 20-min intervals and subsequently at 5-min intervals as setting approached. Because repeated indentation may potentially influence the specimen surface during setting, the indentation schedule used in the present study should be considered when interpreting the setting-time results. Furthermore, ISO 6876:2025 [14] introduced substantial modifications to the setting-time test, including changes in specimen preparation and indentation timing. Consequently, the setting-time values obtained in the present study should be interpreted within the context of the experimental protocol used and should not be considered directly equivalent to values obtained using the current ISO 6876:2025 procedure. This methodological consideration may also contribute to differences between the present setting-time values and those reported by the manufacturers.

Future studies should investigate the long-term stability of premixed CSSs under extended storage periods and repeated environmental fluctuations encountered during transportation and clinical storage. In addition to conventional physicochemical testing, direct measurements of rheological properties, surface tension, wettability, contact angle, and moisture uptake would help clarify how environmental exposure alters material behavior. Physicochemical and microstructural characterization using techniques such as X-ray diffraction, Fourier-transform infrared spectroscopy, and scanning electron microscopy may further elucidate whether changes in hydration products, phase composition, or microstructure are associated with the observed alterations in flow and setting behavior. Studies performed according to the current ISO 6876:2025 setting-time protocol are also warranted to determine whether the setting-time patterns observed here are reproduced using the revised methodology.

## 5. Conclusions

Within the limitations of this study, short-term environmental storage conditions, temperature and relative humidity, significantly influenced the flow and setting behavior of premixed CSSs, with material-dependent responses. All tested sealers maintained flow values above the minimum ISO requirement under the nine storage conditions evaluated. These findings indicate that environmental exposure may alter the handling-related properties of premixed CSSs without necessarily compromising their compliance with the ISO flow requirement. Because the observed responses varied among materials, clinicians should follow product-specific manufacturer recommendations and avoid unnecessary exposure to extreme storage conditions. Further studies using extended storage periods, direct physicochemical characterization, and the current ISO 6876:2025 setting-time protocol are warranted.

## Figures and Tables

**Table 1:** Composition, manufacturer, and batch numbers of the premixed calcium silicate-based sealers tested in this study.

Product Name	Lot No.	Composition *	Manufacturer
One-Fil	OS59T1323	Calcium silicate compounds, Zirconium oxide, Thickening agents	MEDICLUS
CeraSeal	CLS2401122	Calcium silicates, Zirconium dioxide, 1,3-Propanediol, Thickening agent	META BIOMED
Well-Root ST	WR563100	Calcium aluminosilicate compound, Zirconium oxide, Calcium sulfate dihydrate, Titanium dioxide, Inorganic glass including calcium sodium phosphosilicate, Lubricants	VERICOM
ENDOSEAL MTA	KD231223	Natural pure cement, Zirconium dioxide, Bismuth trioxide, Bentonite clay, N-Methyl-2-pyrrolidone, Hypromellose	MARUCHI

* Composition is based on FDA-registered information.

## Data Availability

The original contributions presented in this study are included in the article. Further inquiries can be directed to the corresponding author.

## Abbreviations

CSSs	Calcium silicate-based sealers
